# SNX19 contributes to neurodegeneration by disrupting the endolysosomal system in neurons

**DOI:** 10.1002/alz.087381

**Published:** 2025-01-03

**Authors:** Liang Ma, Luis Aguirre, Debroah Holstein, Vanesa Estevez, James Lechleiter, Jenny Hsieh, Sudha Seshadri

**Affiliations:** ^1^ University of Texas Health Science Center at San Antonio, San Antonio, TX USA; ^2^ University of Texas at San Antonio, San Antonio, TX USA

## Abstract

**Background:**

SNX19 is a key player in endolysosomal and autophagy pathways, which have been extensively reported in neuronal dysfunction and neurodegenerative diseases. Although genetic and cellular evidence suggests SNX19 contributes to neuropathology, the underlying mechanisms remain unknown. Here, we propose to study the mechanism in aging postmortem brain tissue at single cell level and model SNX19 in human induced pluripotent stem cell (hiPSCs) derived brain organoids.

**Method:**

We collected human postmortem brain dorsolateral prefrontal cortex (DLPFC) single‐cell RNA‐seq data from Religious Orders Study/Memory and Aging Project (ROSMAP N = 48). We obtained two human induced pluripotent stem cells (hiPSCs).

**Result:**

Single‐molecule in situ hybridization experiments found that SNX19 is highly expressed in neurons, particularly excitatory neurons, compared to glia in human postmortem brains. Our single‐cell RNA‐seq data further demonstrated that SNX19 gene expression is significantly associated with neuritic plaques in excitatory neurons in postmortem brains.

Cerebral organoid technology has made it possible to model human neurophysiology and disease with increasing accuracy in human‐derived tissue cultures. We performed advanced CRISPR gene editing in hiPSCs to knockout SNX19. We then differentiated them into 2D neurons and 3D cerebral organoids to evaluate the SNX19 impact. Our preliminary data has shown that SNX19 knockout can increase synaptic markers’ expression in hiPSC‐derived neurons. We observed morphological changes in SNX19 knockout organoids and replicated the synaptic markers’ change in the SNX19 knockout brain organoids.

**Conclusion:**

Our study identified novel AD factors at SNX19 in human postmortem brains and will define its role in AD using human‐derived tissue cultures.

